# PREFACE: In silico pipeline for accurate cell‐free fetal DNA fraction prediction

**DOI:** 10.1002/pd.5508

**Published:** 2019-07-11

**Authors:** Lennart Raman, Machteld Baetens, Matthias De Smet, Annelies Dheedene, Jo Van Dorpe, Björn Menten

**Affiliations:** ^1^ Department of Pathology, Ghent University Ghent University Hospital Ghent Belgium; ^2^ Center for Medical Genetics, Ghent University Ghent University Hospital Ghent Belgium

## Abstract

**Objective:**

During routine noninvasive prenatal testing (NIPT), cell‐free fetal DNA fraction is ideally derived from shallow‐depth whole‐genome sequencing data, preventing the need for additional experimental assays. The fraction of aligned reads to chromosome Y enables proper quantification for male fetuses, unlike for females, where advanced predictive procedures are required. This study introduces PREdict FetAl ComponEnt (PREFACE), a novel bioinformatics pipeline to establish fetal fraction in a gender‐independent manner.

**Methods:**

PREFACE combines the strengths of principal component analysis and neural networks to model copy number profiles.

**Results:**

For sets of roughly 1100 male NIPT samples, a cross‐validated Pearson correlation of 0.9 between predictions and fetal fractions according to Y chromosomal read counts was noted. PREFACE enables training with both male and unlabeled female fetuses. Using our complete cohort (n_female_ = 2468, n_male_ = 2723), the correlation metric reached 0.94.

**Conclusions:**

Allowing individual institutions to generate optimized models sidelines between‐laboratory bias, as PREFACE enables user‐friendly training with a limited amount of retrospective data. In addition, our software provides the fetal fraction based on the copy number state of chromosome X. We show that these measures can predict mixed multiple pregnancies, sex chromosomal aneuploidies, and the source of observed aberrations.

## Abstract 2

What's already known about this topic?

- Cell‐free fetal DNA fraction is an important estimate during noninvasive prenatal testing (NIPT).
- Most techniques to establish fetal fraction require experimental procedures, which impede routine execution.

What does this study add?

- PREFACE is a novel software to accurately predict fetal fraction based on solely shallow‐depth whole‐genome sequencing data, the fundamental base of a default NIPT assay.
- In contrast to previous efforts, PREFACE enables user‐friendly model training with a limited amount of retrospective data.

1

**What's already known about this topic?**

- Cell‐free fetal DNA fraction is an important estimate during noninvasive prenatal testing (NIPT).
- Most techniques to establish fetal fraction require experimental procedures, which impede routine execution.

**What does this study add?**

- PREFACE is a novel software to accurately predict fetal fraction based on solely shallow‐depth whole‐genome sequencing data, the fundamental base of a default NIPT assay.
- In contrast to previous efforts, PREFACE enables user‐friendly model training with a limited amount of retrospective data.

## 2 INTRODUCTION

Noninvasive prenatal testing (NIPT) has evolved into an important routine clinical practice. Numerous variations on experimental and in silico procedures have been shown to reliably detect fetal chromosomal aneuploidies, mostly concerning trisomies 13, 18, and 21.^1^, ^2^, ^3^, ^4^, ^5^ The accuracy of NIPT seems high; however, as fetal fragments are scattered throughout a more abundant maternal background in blood plasma, individual performance highly depends on the fraction of fetal‐derived cell‐free DNA (FF). Indeed, the minimal FF for reporting unilateral conclusions has often been debated to be 4%, though lower limits are alleged.^6^, ^7^, ^8^

Several fetal gender‐independent methodologies have been described to assess FF. Prior parental genomic information often facilitates some of these procedures, as, eg, paternal or maternal homozygous loci that are determined to be partly heterozygous in maternal blood during pregnancy form a precise platform to quantify FF.^9^, ^10^, ^11^ Nonetheless, parental priors are not always obliged: using binomial mixture modeling, fetal and maternal clusters of single nucleotide polymorphisms also reflect FF, yet a higher sequencing depth is required.^12^ Likewise, different inputs, such as molecule size (cell‐free fetal DNA fragments are often shorter) and methylation patterns (some fetal sites are hypermethylated), enable FF prediction.^13^, ^14^, ^15^, ^16^

Routine NIPT is converging towards a cost‐effective recipe, with back‐hand automated computational pipelines expecting mostly single‐end shallow‐depth whole‐genome sequencing data (sWGS; 0.1‐1x coverage) to determine copy number alterations.^17^ Previously discussed FF determining techniques imply the need for additional laboratory steps and/or (currently) nonfeasible deep sequencing. Therefore, a handful tools have been developed to predict FF based on exclusively sWGS data. The copy number state of the X chromosome, and especially the number of observed Y chromosomal reads, form popular foundations to calculate FF—here, these are referred to as fetal fraction based on chromosome X (FFX) and fetal fraction based on chromosome Y (FFY), respectively.^18^, ^19^ Unfortunately, they are only informative for male fetuses. Accordingly, two other approaches have been described to predict FF, without relying on the gonosomes. One of these exploits nucleosome positions, hypothesizing that shorter fetal fragments are caused by differential nucleosome packaging.^20^ The spatial distribution of mapped reads should represent FF; however, the reported performance of the predictive model seems rather unsatisfactory.^19^ Finally, SeqFF, which uses a model designed directly on bin‐wise copy number features of more than 25 000 pregnant women, reports accurate FF determination, with a Pearson correlation between predictions and FFY of 0.932.^21^ The inventors state that cell‐free fetal and maternal fragments are not uniformly distributed across the human reference genome: small differences in local read counts are predictive for FF. Aside from the seemingly excessive number of required male training samples, the software does not provide a training option. Therefore, users are restricted to a pretrained alternative. Because of inevitable differences in laboratory and computational procedures between training and test cases, the correlation is expected to be lower than what is claimed.

Applying similar biological principles as used by SeqFF, we set out to develop PREdict FetAl ComponEnt (PREFACE), a software that enables model training, utilizing a limited amount of data, which includes unlabeled female samples to maximize the input. The semisupervised pipeline operates an initial unsupervised phase, in the form of a principle component analysis (PCA), and a subsequent supervised step, where a neural network (NN) weighs the computed principle components (PCs) to model fetal‐induced variance.

## 3 MATERIALS AND METHODS

### 3.1 Library preparation and sequencing

Blood samples were collected in 10‐mL cell‐free DNA BCT tubes (Streck) or PAXgene Blood DNA Tubes (Qiagen). Within 24 hours after collection, plasma isolation was executed by centrifugation (4°C; 10 minutes at 1600 *g*; 10 minutes at 16 000 *g*, or 15 minutes at 1900 *g*, respectively). The supernatant was transferred to a new tube and cfDNA was extracted from 3.5‐mL plasma using the Maxwell RSC ccfDNA Plasma Kit (Promega), following the manufacturer's instructions.

Using 25 μL of cfDNA, library preparation was executed on a Hamilton Star liquid handler using the NEXTflex Cell Free DNA‐Seq Library Prep Kit (Bioo Scientific) and NEXTflex DNA Barcodes (Bioo Scientific). After pooling, cluster generation and sequencing were completed by respectively a cBot 2 and HiSeq 3000 system (Illumina). The minimal number of reads (single‐read; 50‐cycle mode) per sample was set to 15 million.

### 3.2 Copy number profiling

Raw reads were mapped by Bowtie 2 onto human reference genome GRCh38 (and GRCh37, for SeqFF compliance), using the *fast‐local* flag.^22^ Biobambam's bamsormadup was used to mark duplicate reads and to sort resulting bam files.^23^ Indexing was executed by SAMtools.^24^ To reliably deduce normalized bin‐wise log_2_ ratios from sWGS data, we preferred WisecondorX, considering it yields superior copy number profiles, as shown by our group in earlier work.^25^ These ratios represent the relation between the observed (numerator) and expected (denominator) number of reads, the latter matching the diploid state. Since these values are subject to Gaussian noise, a resolution of 100 kb was selected to yield reasonable noise levels in function of the obtained number of reads (20 534 289 ± 5 662 927). Regions without resulting information were interpreted as loci of undeterminable copy number, as defined by WisecondorX.

### 3.3 NIPT cohort

From December 2017 until September 2018, 5629 NIPT experiments were routinely executed at the Center for Medical Genetics Ghent, of which 5572 passed quality filtering, including 177 echographically confirmed twins, one triplet, and 14 fetuses with confirmed trisomies for chromosome 13, 18, or 21 by chorionic villus sampling or amniocentesis. All analyses were applied to this set, with the exception of the actual model training and subsequent cross‐validation. For these parts, we defined a second set (n_female_ = 2468, n_male_ = 2723) after applying an additional filter: exclusively gender‐annotated single and same‐gender multiple pregnancies were allowed, where five more male samples, suspected of having sex aberrations according to differences in FFY and SeqFF computations, were excluded.

### 3.4 Response variable FFY

For male fetuses, the FF is linearly proportional to the read depth‐corrected mean number of observed Y reads (*Y*_*NIPT*,*male*_). In the formula below, the prior or naive FFY is interpreted as a *Y*_*NIPT*,*male*_ observation between the median of a set of male liquid biopsies (LBs) $\tilde{Y_{\mathrm{LB},\text{male}}}$ (FFY = 100%) and female background noise $\tilde{Y_{\text{NIPT},\text{female}}}$ (FFY = 0%). For female fetuses, the prior FFY is set to 0.

(1) $${\mathrm{FFY}}_{\text{prior},\text{male}}=\frac{Y_{\text{NIPT},\text{male}}-\tilde{Y_{\text{NIPT},\text{female}}}}{\tilde{Y_{\mathrm{LB},\text{male}}}-\tilde{Y_{\text{NIPT},\text{female}}}}$$

(2) $${\mathrm{FFY}}_{\text{prior},\text{female}}=0$$

As previously reported, masking the Y chromosome prior to calculating FFY increases the precision.^18^, ^19^ We took this concept one step further by creating a model that provides a weighted selection of the most appropriate set of Y windows. This way, a large increase in power to separate males from females was noted. We believe hypervariable FF‐unrelated bins are down‐weighted, forming a supposed overall more accurate FFY. A general linear model with lasso regularization (λ = 1e^−4^) was selected, using the read depth‐normalized number of reads at 5 kb Y bins as explanatory parameters, and the prior FFY as a response variable (Figure S1). The fitted model parameters were retrieved to infer a final FFY, as shown below.

(3) $${\mathrm{FFY}}_{\text{final}}=\beta_{0}+\beta_{1}y_{1}+\ldots +\beta_{n}y_{n}$$

Above, *β*_0_ is the intercept, *β*_*k*_ indicates the beta estimate for bin *k*, whereas *y*_*k*_ represents the observed normalized number of reads at the same locus. Chromosome Y has *n* bins (n = 11 447). Note that *FFY*_*final*_ was calculated using a cross‐validation strategy: different models were trained to circumvent overlap between train and test cases. An overall model determined that 10.76% of chromosome Y remained available for FFY determination (*β*_*k*_ ≠ 0). The Pearson correlation between the prior and final FFY was 0.985 for male fetuses.

### 3.5 PREFACE method

To maximize training input, PREFACE uses a combination of unsupervised (applicable to all NIPT samples) and supervised learning (applicable to samples with known FF, being all male fetuses in our case). The explanatory variables comprise all autosomal bins for which a log_2_ ratio could be derived. Note that an exception holds: loci at chromosomes 13, 18, and 21 are excluded—this is because these chromosomes might be wrongly estimated as highly related to FF due to the presence of fetal aneuploidies in the training set.

#### 3.5.1 Unsupervised learning

Between observations (samples), some explanatory variables (bins) are expected to be codependent as a result of inter alia differing FFs. In other words, nonrandom variance, linked to FF, is thought to be present. PCA is a technique to model the observed variance by orthogonal transformation: the original explanatory variables are converted to new linearly uncorrelated parameters, named PCs.^26^ PCs are ranked in order of importance, meaning each PC explains less variance than its predecessor. The first set of PCs (n_default_ = 50) models a large portion of the nonrandom variance, thus including FF‐induced variance, whereas the remaining PCs mostly map naturally occurring Gaussian noise, as a result of the original binomial read count distribution.^27^ The computed PCA rotations, based on all NIPT samples, enable us to calculate the most important PCs for exclusively cases with known FF. This latter set is further processed in the supervised phase.

#### 3.5.2 Supervised learning

As stated, PCA presumably separates Gaussian noise from other sources of variance. Consequently, a supervised classifier is required to model exclusively FF‐induced variance. We preferred an artificial NN with two hidden layers, using resilient backpropagation with weight backtracking, and the sum of squared errors as a loss function. This black box method weighs parameters (PCs) in function of the response variable (FFY). As machine learning often tends to find the best solution for most cases, rather than for all, predictions and FFY values are slightly “slanted” relative to each other. A default slope and intercept extracted from a linear model corrects for this tendency.

### 3.6 PREFACE software

**Figure 1:**
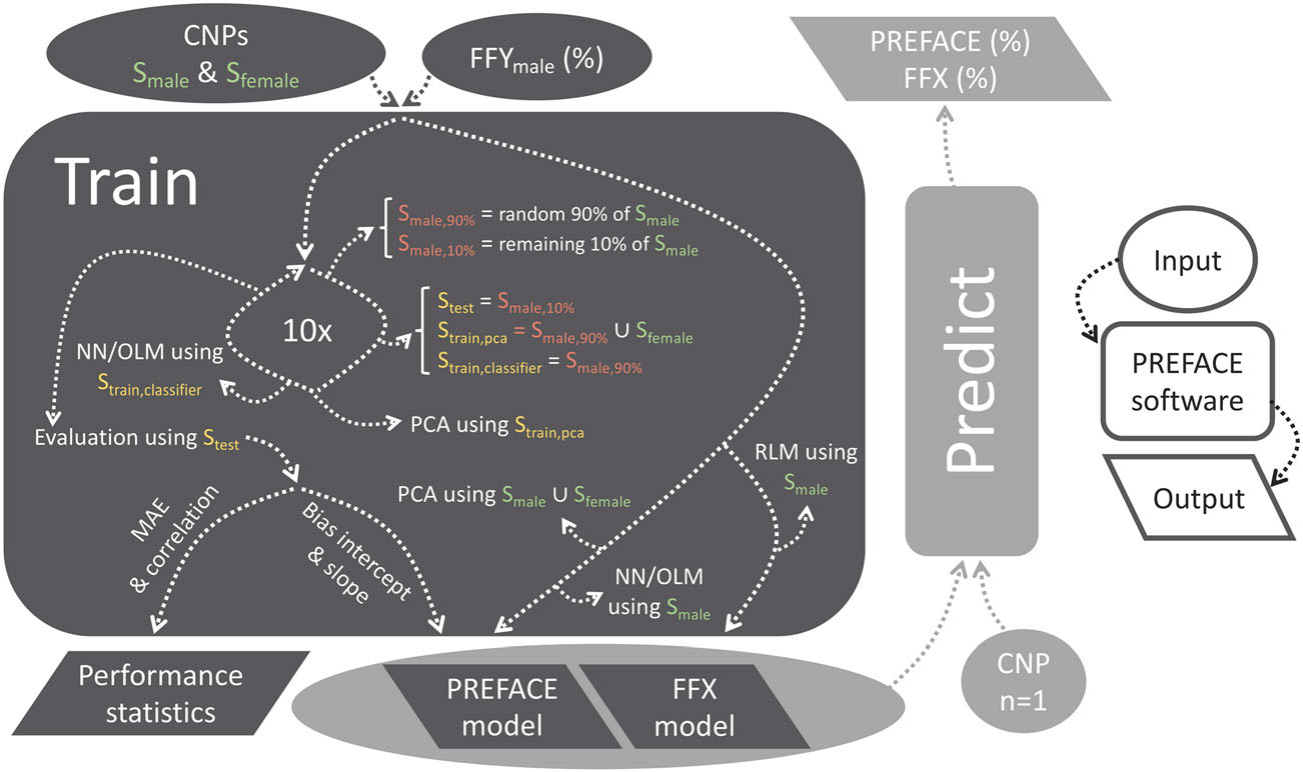
Schematic representation of the PREFACE software. The “train” component (dark grey) accepts NIPT copy number profiles from both male and female fetuses. A predictive model is generated using all provided samples. To gain insight in the performance of this model, 10‐fold cross‐validation is executed in addition. The “predict” component (light grey) makes predictions by applying the trained model to a supplied copy number profile. Abbreviations: CNP, copy number profile; FFX, fetal fraction based on chromosome X; FFY, fetal fraction based on chromosome Y; MAE, mean absolute error; NIPT, noninvasive prenatal testing; NN, neural network; OLM, ordinary linear model; PCA, principal component analysis; PREFACE, PREdict FetAl ComponEnt; RLM, robust linear model; S, set [Colour figure can be viewed at wileyonlinelibrary.com]

The PREFACE software, written in R, is divided in two large components: one for training and one for predicting (Figure 1). It is available at https://github.com/CenterForMedicalGeneticsGhent/PREFACE.

#### 3.6.1 Training

When feeding the train module copy number profiles in combination with FFY measurements from male fetuses, a model is created as described above. Since NNs can experience convergence problems, and as they were noted to be less performant on small training sets, an ordinary linear model (OLM) can alternatively be selected as a classifier. Performance statistics are derived from a 10‐fold cross‐validation technique: 10% of male samples are iteratively ignored during training, followed by evaluating the correlation and mean absolute error between FFY and predictions in the left‐out test set. In addition, PREFACE fits a robust linear model (RLM) between the overall ratio (observed/expected number of reads) of chromosome X and FFY, enabling FFX calculations. A robust technique was favored to sideline (mosaic) (sub)chromosomal maternal deviations during training.

#### 3.6.2 Predicting

The predict component accepts a trained model and a NIPT copy number profile. Bins without information are replaced by interpolated mean training values. PREFACE transforms bin‐wise values to PCs using the PCA rotations and subsequently outputs the FF according to the NN. The robust least squares fit is applied to chromosome X's ratio to retrieve FFX.

## 4 RESULTS

### 4.1 The PREFACE modeling strategy proves to be powerful

Two important aspects should be evaluated to assess the competence of our approach: the tightness of a relation is given by the Pearson correlation (*r*), whereas the agreement between two methods can be explored by both visual interpretation—by use of a least squares fit and an identity line—and the mean absolute error (MAE).^28^

The PREFACE software was executed four times across pairwise combinations between two data sets (male‐only NIPT samples; all NIPT samples) and two classifiers (OLM; NN). In comparison, a state‐of‐the‐art supervised elastic net was optimized in accordance to Friedman et al, therefore exclusively trained with male fetuses.^29^

#### 4.1.1 Males

**Figure 2:**
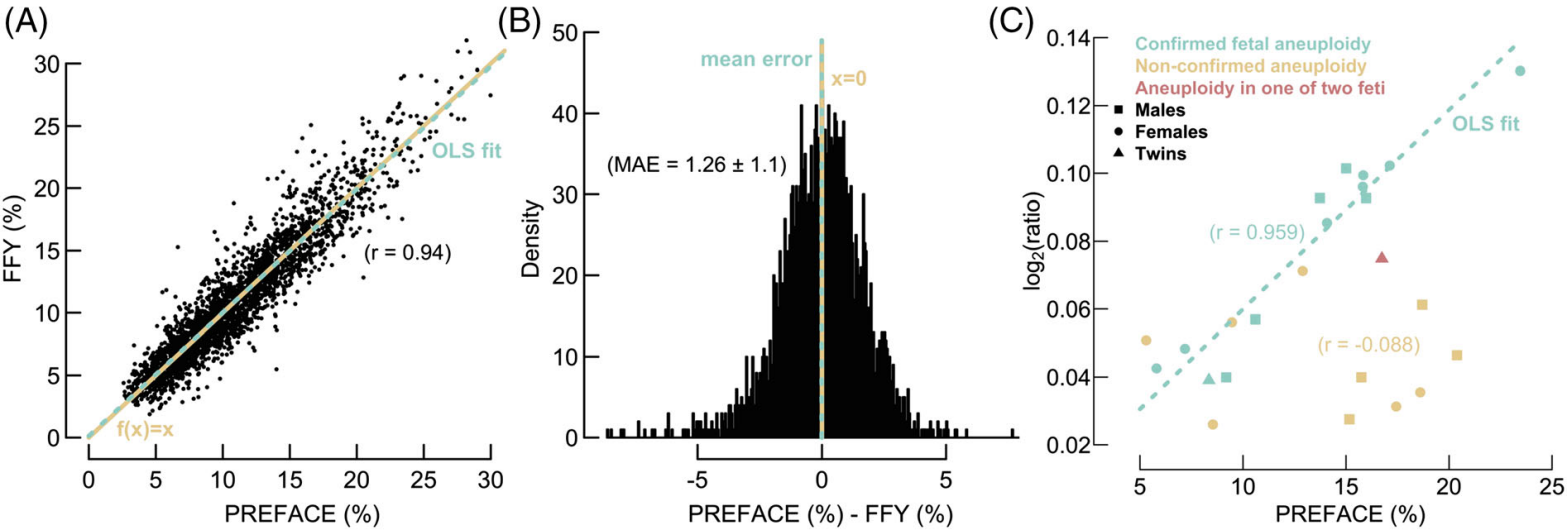
Performance evaluation of the PREFACE method. A, A scatter plot reveals highly correlated (r) FFY and PREFACE predictions. Moreover, the OLS fit largely covers the identity line. B, A histogram visualizes normally distributed errors centered around 0 and a low MAE between predictions and FFY. C, Scattered symbols indicate reported NIPT samples with aneuploidies. The dotted line represents an OLS fit between the PREFACE values and the mean log_2_ ratio of the corresponding structural validated events. Where confirmed aberrations are highly concordant to FF predictions, nonconfirmed aneuploidies are randomly scattered. Abbreviations: OLS, ordinary least squares; MAE, mean absolute error; FF, fetal fraction; FFY, fetal fraction based on chromosome Y; NIPT, noninvasive prenatal testing; PREFACE, PREdict FetAl ComponEnt [Colour figure can be viewed at wileyonlinelibrary.com]

Cross‐validation indicates that PREFACE is superior to a traditional elastic net (Table 1). The NN, default in PREFACE, performs generally better than the optional OLM. Although the classifiers are trained with male fetuses only, the inclusion of females during the unsupervised phase significantly improves performance: the correlation between predictions and FFY rises from 0.926 to 0.94, while the MAE drops 0.18 units—statistics emerging from the NN (Figure 2A,B). Indeed, adding female samples (or in general, adding more samples) enables the PCA algorithm to explain a larger proportion of (nonrandom) variance in its most important PCs (Figure S2). Although NNs perform generally better, users can opt for an OLM instead, as these tend to be more reliable on smaller data sets (Figure S3). For sets of roughly 1100 male samples, a correlation of 0.9 is reached.

**Table 1:** Cross‐validation to compare combinations of classifiers and training sets

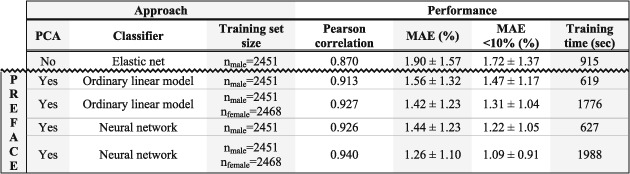

*Note*. Next to four setups applicable to the PREFACE software, a traditional elastic net was optimized to support comparison. A model initialized with default arguments, trained using NIPT samples from both male and female fetuses, enables the most accurate predictions, measured by Pearson correlation and MAE. The MAE for the lowest FFs (<10%) is shown separately. Although multicore processing is optional, timing was performed on a system equipped with a 2.3 GHz Intel Core i5 processor using only a single thread. Abbreviations: MAE, mean absolute error; FF, fetal fraction; NIPT, noninvasive prenatal testing; PCA, principal component analysis; PREFACE, PREdict FetAl ComponEnt.

#### 4.1.2 Females

Since NIPT samples from female fetuses lack independent FF measurements, PREFACE values were compared with SeqFF predictions, an approach proven to be applicable to female cases. Two major conclusions could be drawn. First, for males, the correlation between FFY and SeqFF predictions is “only” 0.887, lower than the reported 0.932, thus presumably caused by experimental differences between the pretrained SeqFF model and FFY (Figure S4a).^21^ Moreover, the least squares fit is considerably less steep than the identity line, showing that SeqFF claims mostly higher FFs. Second, applying the female samples to a male‐only PREFACE model yields a correlation with SeqFF of 0.895 (Figure S4b). As expected, a similar yet inverse inconsistency with the identity relation is retrieved, validating PREFACE's applicability to female fetuses.

#### 4.1.3 Fetal fraction based on chromosome X

The relation between FFX and FFY seems trivial. Therefore, the PREFACE software solely fits an RLM to the provided male fetuses without executing cross‐validation. A weighted correlation as high as 0.971 supports this approach (Figure S5).^30^ Extreme outliers are caused by (mosaic) (sub)chromosomal maternal rearrangements, illustrating the need for a robust model.

### 4.2 There is a strong correlation between FF predictions and confirmed aneuploidies

Throughout the NIPT cohort, 14 fetuses were reported with confirmed aneuploidies. These involve two cases with Patau syndrome (trisomy 13), one with Edward syndrome (trisomy 18), and 11 with Down syndrome (trisomy 21). Unconfirmed aneuploidies (after amniocentesis) include, eg, nonviable trisomies 7, 14, and 20, representing aberrations that are likely mosaicisms confined to the placenta. Another reported abnormality, concerning trisomy 21, was shown to be unrelated to the fetus by amniocentesis.

Fetal‐derived nonmosaic aberrations are expected to have an amplitude proportional to the FF (1,6,7). Hence, prior to the execution of an invasive assay, predictions on FF suggest the source of a potential aneuploidy. This is shown by a compelling concordance between the mean log_2_ ratio of confirmed whole‐chromosome duplications and predictions of *r* = 0.959, additionally indicating PREFACE's accuracy (Figure 2C). Where the amplitudes of fetal abnormalities are positioned to expectation, defined as in Adalsteinsson et al, nonfetal observations are randomly scattered (Figures S6 and S7).^31^ Here, the difference between the expected FF (based on confirmed aberrations) and predicted FF (according to PREFACE) is characterized by a standard deviation of 1.92%.

### 4.3 PREFACE empowers gender prediction in multiple pregnancies

Besides single pregnancies, the NIPT cohort includes 177 twins, established through ultrasonography. The ratio between FFY and true FF naturally provides information about the gender of each fetus: two males are theoretically characterized by a ratio of 1; while with female twins, this measure amounts to 0, whereas for mixed pregnancies, a close‐to 0.5 ratio is expected.

**Figure 3:**
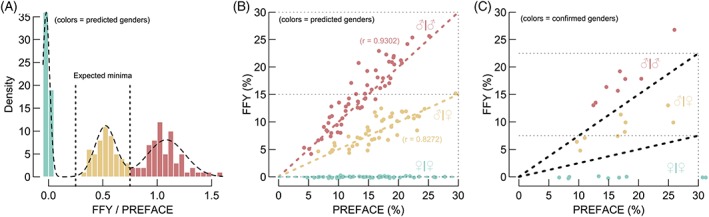
Gender prediction in twins. A, Including twins without confirmed genders, a Gaussian mixture model, expecting three components, fits the FFY/PREFACE density distribution well. The expected local minima (at one‐fourth and three‐fourth) represent cutoffs to predict fetal gender. B, A scatter visualization plots the PREFACE predictions in function of FFY. Colors are defined by previous cutoffs. Thick dotted lines represent the theoretical expectation. Pearson correlations (*r*) are given. C, Evaluation of this method using confirmed (by birth) twin genders. Thick dotted lines represent the cutoffs from (A). Colors are defined by actual gender. Abbreviations: FFY, fetal fraction based on chromosome Y; PREFACE, PREdict FetAl ComponEnt [Colour figure can be viewed at wileyonlinelibrary.com]

Our cohort contains both confirmed (by birth) and unconfirmed twin genders. The density distribution of the ratio between FFY and FF intrinsically represents the ability to distinguish different combinations of genders. Using Gaussian mixture modeling, three distinct peaks are retrieved across twins lacking gender confirmation (Figure 3A). This suggests that female twins can be categorized with high accuracy, yet, discriminating male‐male from male‐female twins remains difficult for pregnancies with low FF (Figure 3B). Finally, a similar visualization, holding validated genders, does confirm the reliability of this technique (Figure 3C).

### 4.4 PREFACE indirectly hints towards potential sex aneuploidies

With PREFACE, FFY, and FFX, three methods have been presented to establish FF. A consequence of adopting these estimates—next to what has already been discussed—is the inherent information on sex aneuploidies they potentially reveal. Sex aneuploidies were until now not reported by our institution; therefore, none are confirmed, meaning this final section is purely indicative and further experimental validation is warranted.

A dual modeling strategy was developed. First, by simultaneously comparing both FFX and FFY to PREFACE predictions, the power to distinguish genders increases.

(4) $$\text{Density}\;1_{i}=\sum_{j=0}^{i}\left(\frac{{\mathrm{FFY}}_{j}+{\mathrm{FFX}}_{j}}{{\text{PREFACE}}_{j}*2}\right)$$

Second, most frequent sex aneuploidies, including Turner (X), triple‐X (XXX), Klinefelter (XXY), and XYY syndrome, are theoretically captured by directly subtracting FFY with FFX, independent from gender.

(5) $$\text{Density}\;2_{i}=\sum_{j=0}^{i}\left({\mathrm{FFY}}_{j}-{\mathrm{FFX}}_{j}\right)$$

**Figure 4:**
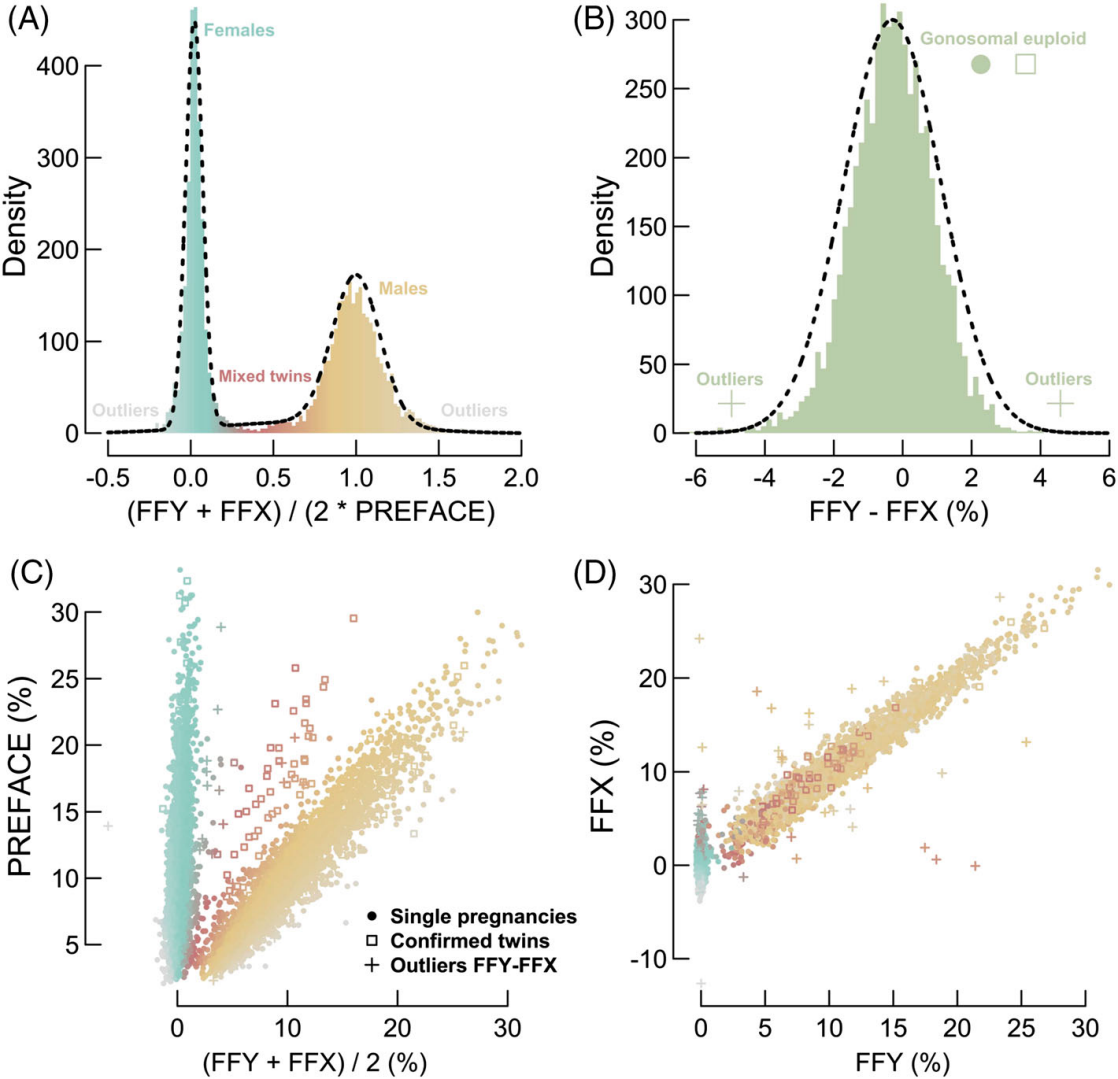
Modeling of FFY, FFX, and PREFACE measures to predict mixed twins and sex aneuploidies. A, Density Gaussian mixture modeling to map fetal gender. The color gradient is linearly assigned in accordance to the component's means. Outliers are shown in grey. B, A Gaussian distribution is fitted to appoint outliers, where the latter is defined as such once it deviates with more than 3 standard deviation units from the mean. C, *FFY‐FFX‐PREFACE viewpoint 1*. Colors are defined by (A); dots and squares represent confirmed single and twin pregnancies, respectively; plusses overrule previous symbols, as determined by (B). As expected, it is notable that red symbols are highly enriched with twins, especially for higher FFs. D, *FFY‐FFX‐PREFACE viewpoint 2*. Colors and symbols are defined in analogy to (C). Outliers likely correspond to maternal events or fetal sex aneuploidies. Abbreviations: FF, fetal fraction; FFX, fetal fraction based on chromosome X; FFY, fetal fraction based on chromosome Y; PREFACE, PREdict FetAl ComponEnt [Colour figure can be viewed at wileyonlinelibrary.com]

Eight FFX outliers (less than −40%; greater than 40%), caused by maternal aberrations, were removed prior to fitting Gaussian (mixture) models to analytically describe the density distributions, expecting three (males, females, and mixed twins) and one component(s), respectively (Figure 4A,B). Optimally, the results are presented in a three‐dimensional all‐inclusive figure, plotting FFY, FFX, and PREFACE values along its axes (File S1). Here, we opted to visualize the results in accordance to two preferred viewpoints (Figure 4C,D). It is notable that confirmed twins are highly enriched in the middle Gaussian component of *Density* 1: these are mixed twin pregnancies. In total, 39 (0.71%) cases significantly deviate from the healthy FFY‐FFX trend. The majority of these likely concern (mosaic) maternal events and a few suspected subchromosomal aberrations. However, four XXY, two XYY, one XXX, and none X fetuses seem to be present when evaluating the FFX‐FFY outliers in function of the PREFACE predictions (Figure S8). Worth saying, these numbers largely correspond to reported incidence.^32^, ^33^, ^34^, ^35^

## 5 DISCUSSION

Recent technological advancements improved genetic testing dramatically. While the economic feasibility of whole‐genome sequencing keeps progressing, the accuracy of fetal aneuploidy detection is at an ever‐high: sWGS studies commonly report near 0.99 sensitivity/specificity for Patau, Edward, and Down syndrome detection.^8^, ^36^ As a consequence, noninvasive screening is no longer confined to high‐risk groups but is gradually more generally executed.

Large NIPT turnovers produce an abundance of retrospective useful data. One interesting application enabled by these quantities is machine learning, as, eg, FF, a particularly important figure during testing, can be estimated based on copy number data. Predictive models are ideally trained with in‐house profiles to suppress between‐laboratory procedural bias. Notwithstanding, sufficient data are frequently present at these institutions; to date, an accurate automated learning software does not exist. Therefore, we developed PREFACE, a user‐friendly tool to model and predict FF without the necessity of prior mathematical know‐how on predictive modeling. The inclusion of unlabeled samples for training, which significantly contributes to an increased overall performance, introduces another novelty to this field.

Using less than 5000 training samples, predictions made by PREFACE were highly concordant to FFY, indicated by a Pearson correlation of 0.94. To our knowledge, starting from sWGS data only, no software has been reported to perform better. Next to traditional cross‐validation, PREFACE was evaluated by SeqFF comparison (for female fetuses); by density Gaussian mixture modeling across twins; and by aneuploid fetuses, where the log_2_ ratio of confirmed events was found to be highly concordant with FF (*r* = 0.959).

Since the SeqFF trend was not in satisfying agreement with FFY (SeqFF claims mostly higher FFs), one could wonder which of both variables is truly biased. Accordingly, not presented in the results, we computed FFY and FFX for six liquid biopsies and six lymphocyte‐extracted genomic DNA samples, obtaining percentage estimations ranging within {98, 103} and {−1, 1} for males and females, respectively. Moreover, PREFACE's model, trained on FFY, yields predictions that are conform with confirmed trisomies (Figures S6 and S7). The pretrained SeqFF model is therefore more likely to be biased rather than FFY.

The success of the modeling approach is thought to involve three main pillars. First, we believe that the FFY measure, although hard to prove, is accurate. Masking parts of chromosome Y prior to predicting FF has been cited to increase correctness.^18^, ^19^ Due to sequence similarities with other chromosomes (eg, the pseudoautosomal region) and technological limitations of short‐read mapping (repeats, variable regions of mappability, GC content, etc), numerous Y loci are indeed ambiguous.^37^, ^38^ Instead of solely categorizing bins as informative and noninformative, we reasoned that the informative bins also differ in their “level of male specificity,” thereby encouraging the idea of a bin‐wise weighted contribution to FFY. Second, read count normalization was executed by WisecondorX, a sophisticated within‐sample normalization procedure, which supposedly delivers superior profiles.^25^ And last but not least, the nature of the modeling strategy maximizes training input by allowing unlabeled samples.

Gonosomal aberrations are theoretically exposed during NIPT in a similar way as any other aneuploidy. Nevertheless, the specificity is reported to be much lower in comparison with traditional screening of chromosomes 13, 18, and 21, especially for monosomy X.^39^, ^40^, ^41^ Ethical issues on reporting these sometimes nonsevere abnormalities aside, the incorporation of FF in statistical outcome—which is generally not done with, eg, the popular *z*‐score approach—does improve performance.^42^, ^43^ Indeed, our study was concluded by revealing that 0.71% of all NIPT samples significantly differed from the healthy gonosomal trend; however, when evaluating these outliers in relation to predicted FF, only a few truly met the requirements to suffice as being potentially sex aneuploid.

The convenience by which PREFACE could be implemented in existing NIPT pipelines seems undeniable: a copy number profile, the fundamental base of an assay, is singly requisite as input. This paper extensively demonstrates the practical value of accurate FF estimations on real data collected over the course of nine months. We believe PREFACE and the elaborated FF methodologies could be useful to many NIPT laboratories, evidentially motivating this work.

## CONFLICT OF INTEREST

None declared.

## FUNDING SOURCES

This work was supported by Bijzonder Onderzoeksfonds (BOF), Ghent University, in the form of a doctoral research grant (ID BOF.STA.2017.0002.01 to L.R.).

## ETHICS STATEMENT

This study was conducted according to the guidelines of the Ethics Committee at Ghent University Hospital (ID 2004/094).

## DATA AVAILABILITY STATEMENT

The data that support the findings of this study are available on request from the corresponding author. These are not publicly available due to privacy and ethical restrictions.

## Supporting information


**Figure S1.** **A general regularized linear model to calculate FFY.** (**a**) The beta estimates of the model show which set of bins is suitable to calculate FFY. As expected, the large majority of variables that passed parameter selection by lasso regularization correspond to positive beta values. Three randomly selected male specific genes are shown to indicate the biological sense behind this procedure. (**b**) The FFY measure splits male and female fetuses in two clear distinct groups, where female cases barely differ from 0. The ‘original other’ category depicts multiple pregnancies and ‘gender‐uncertains’.

**Figure S2.** **The proportion of explained variance at principal components (PCs).** PCs are ranked to importance. Both the PCs and the proportion of explained variance is pictured in log‐scale. Including more samples enables the principal component analysis (PCA) algorithm to model a larger proportion of variance within the first set of PCs (1 until 50). Note that ideally, the selected number of features separates the first ‘random’ phase from the subsequent ‘non‐random’ phase, as shown. Including more ‘non‐random PCs’ barely impacts performance, yet it could lead to convergence problems during NN training.

**Figure S3.** **Cross‐validation statistics in function of training set size.** A randomly selected validation set was evaluated across a sequence of 40 male‐only models. (**a**) Ordinary linear models (OLMs) are more stable for smaller sets, whereas neural networks (NNs) start to outperform OLMs with rising training set size, as shown by Pearson correlation (r). (**b**) A similar conclusion is made for the mean absolute error (MAE). Dotted lines represent 95% confidence intervals.

**Figure S4.** **Scatter plot comparison between FFY, SeqFF and PREFACE measures.** (**a**) The FFY metric for male fetuses is shown in function of SeqFF. The ordinary least squares (OLS) fit does not cover the identity line, meaning SeqFF predictions are probably biased. (**b**) SeqFF values in function of PREFACE predictions across females. As expected, a similar yet inverse type of bias is noted. Correlations (r) are highly similar, validating PREFACE's applicability to female fetuses.

**Figure S5.** **Calculating FFX in function of FFY.** (**a**) A robust least squares (RLS) fit results in a significant weighted correlation (wr) of 0.971. The extreme outliers are caused by maternal rearrangements at chromosome X. (**b**) A simple intercept and slope extracted from the RLS fit suffices to compute FFX.

**Figure S6.** **Partly visualized copy number profiles from fetuses with confirmed aneuploidies.** Reported trisomies are marked in red. A dotted slightly darker red line represents the mean amplitude of the corresponding aberration, whereas the yellow line indicates the expected value according to PREFACE's prediction. Observed aberrations are consequently close to expectation.

**Figure S7.** **Partly visualized copy number profiles from fetuses with unconfirmed aneuploidies.** Reported trisomies are marked in red. A dotted slightly darker red line represents the mean amplitude of the corresponding aberration, whereas the yellow line indicates the expected value according to PREFACE's prediction. Observed aberrations are often lower than PREFACE values, suggesting placental mosaicisms.

**Figure S8.** **Discovering sex aneuploidy suspects.** The ratio between FFY and PREFACE values is shown in relation to the ratio between FFX and PREFACE. Colors and symbols are assigned as in Figure 4. The surface of the symbols is proportional to the fetal fraction (FF), and thereby the relevance of the NIPT assay. Ellipse size is defined by the overall observed standard deviations within males and females: these should hold 95% of fetal aneuploidies, expressed as plusses in addition. The arrow points to a couple of female fetuses with similarly elevated FFX and FFY, potentially involving vanishing twins.

**File S1.** **Interactive three‐dimensional FFY‐FFX‐PREFACE plot.** HTML should be interpretable by any browser. Dragging results in figure movement, whereas scrolling can be used to zoom. Axes correspond to FFX (x‐axis), FFY (y‐axis) and PREFACE (z‐axis) values. Expected positions for fetal aneuploidies and mixed twins are indicated by straight lines. Colors and symbols are assigned as in Figure 4.

Supporting info item
