# Supplementary material for: PREFACE: In silico pipeline for accurate cell‐free fetal DNA fraction prediction
Source: Prenat Diagn. 2019 Jul 11;39(10):925–33. doi: 10.1002/pd.5508 (PMC6771918; doi:10.1002/pd.5508)
Supplement: Supplementary file 10 — Supporting info item [file PD-39-925-s010.docx]

**Supporting information**

**Figure S1. A general regularized linear model to calculate FFY.** (**a**) The beta estimates of the model show which set of bins is suitable to calculate FFY. As expected, the large majority of variables that passed parameter selection by lasso regularization correspond to positive beta values. Three randomly selected male specific genes are shown to indicate the biological sense behind this procedure. (**b**) The FFY measure splits male and female fetuses in two clear distinct groups, where female cases barely differ from 0. The ‘original other’ category depicts multiple pregnancies and ‘gender-uncertains’.

**Figure S2. The proportion of explained variance at principal components (PCs).** PCs are ranked to importance. Both the PCs and the proportion of explained variance is pictured in log-scale. Including more samples enables the principal component analysis (PCA) algorithm to model a larger proportion of variance within the first set of PCs (1 until 50). Note that ideally, the selected number of features separates the first ‘random’ phase from the subsequent ‘non-random’ phase, as shown. Including more ‘non-random PCs’ barely impacts performance, yet it could lead to convergence problems during NN training.

**Figure** **S3. Cross-validation statistics in function of training set size.** A randomly selected validation set was evaluated across a sequence of 40 male-only models. (**a**) Ordinary linear models (OLMs) are more stable for smaller sets, whereas neural networks (NNs) start to outperform OLMs with rising training set size, as shown by Pearson correlation (r). (**b**) A similar conclusion is made for the mean absolute error (MAE). Dotted lines represent 95% confidence intervals.

**Figure S4. Scatter plot comparison between FFY, SeqFF and PREFACE measures.** (**a**) The FFY metric for male fetuses is shown in function of SeqFF. The ordinary least squares (OLS) fit does not cover the identity line, meaning SeqFF predictions are probably biased. (**b**) SeqFF values in function of PREFACE predictions across females. As expected, a similar yet inverse type of bias is noted. Correlations (r) are highly similar, validating PREFACE’s applicability to female fetuses.

**Figure S5. Calculating FFX in function of FFY.** (**a**) A robust least squares (RLS) fit results in a significant weighted correlation (wr) of 0.971. The extreme outliers are caused by maternal rearrangements at chromosome X. (**b**) A simple intercept and slope extracted from the RLS fit suffices to compute FFX.

**Figure S6. Partly visualized copy number profiles from fetuses with confirmed aneuploidies.** Reported trisomies are marked in red. A dotted slightly darker red line represents the mean amplitude of the corresponding aberration, whereas the yellow line indicates the expected value according to PREFACE’s prediction. Observed aberrations are consequently close to expectation.

**Figure S7. Partly visualized copy number profiles from fetuses with unconfirmed aneuploidies.** Reported trisomies are marked in red. A dotted slightly darker red line represents the mean amplitude of the corresponding aberration, whereas the yellow line indicates the expected value according to PREFACE’s prediction. Observed aberrations are often lower than PREFACE values, suggesting placental mosaicisms.

**Figure S8. Discovering sex aneuploidy suspects.** The ratio between FFY and PREFACE values is shown in relation to the ratio between FFX and PREFACE. Colors and symbols are assigned as in Figure 4. The surface of the symbols is proportional to the fetal fraction (FF), and thereby the relevance of the NIPT assay. Ellipse size is defined by the overall observed standard deviations within males and females: these should hold 95% of fetal aneuploidies, expressed as plusses in addition. The arrow points to a couple of female fetuses with similarly elevated FFX and FFY, potentially involving vanishing twins.

**File S1. Interactive three-dimensional FFY-FFX-PREFACE plot.** HTML should be interpretable by any browser. Dragging results in figure movement, whereas scrolling can be used to zoom. Axes correspond to FFX (x-axis), FFY (y-axis) and PREFACE (z-axis) values. Expected positions for fetal aneuploidies and mixed twins are indicated by straight lines. Colors and symbols are assigned as in Figure 4.
